# Production of Biodegradable Palm Oil-Based Polyurethane as Potential Biomaterial for Biomedical Applications

**DOI:** 10.3390/polym12081842

**Published:** 2020-08-17

**Authors:** Fang Hoong Yeoh, Choy Sin Lee, Yew Beng Kang, Shew Fung Wong, Sit Foon Cheng, Wei Seng Ng

**Affiliations:** 1 Department of Pharmaceutical Chemistry, School of Pharmacy, International Medical University, Bukit Jalil, Kuala Lumpur 57000, Malaysia; adelineyfh90@gmail.com (F.H.Y.); yewbeng_kang@imu.edu.my (Y.B.K.); 2 Department of Pathology, School of Medicine, International Medical University, Bukit Jalil, Kuala Lumpur 57000, Malaysia; shewfung_wong@imu.edu.my; 3 Unit of Research on Lipids (URL), Department of Chemistry, Faculty of Science, University of Malaya, Kuala Lumpur 50603, Malaysia; sfcheng@um.edu.my (S.F.C.); ng_ws@topglove.com.my (W.S.N.)

**Keywords:** palm oil-based polyester polyol, polyurethane, biodegradable, biocompatible, biomaterial, tissue engineering

## Abstract

Being biodegradable and biocompatible are crucial characteristics for biomaterial used for medical and biomedical applications. Vegetable oil-based polyols are known to contribute both the biodegradability and biocompatibility of polyurethanes; however, petrochemical-based polyols were often incorporated to improve the thermal and mechanical properties of polyurethane. In this work, palm oil-based polyester polyol (PPP) derived from epoxidized palm olein and glutaric acid was reacted with isophorone diisocyanate to produce an aliphatic polyurethane, without the incorporation of any commercial petrochemical-based polyol. The effects of water content and isocyanate index were investigated. The polyurethanes produced consisted of > 90% porosity with interconnected micropores and macropores (37–1700 µm) and PU 1.0 possessed tensile strength and compression stress of 111 kPa and 64 kPa. The polyurethanes with comparable thermal stability, yet susceptible to enzymatic degradation with 7–59% of mass loss after 4 weeks of treatment. The polyurethanes demonstrated superior water uptake (up to 450%) and did not induce significant changes in pH of the medium. The chemical changes of the polyurethanes after enzymatic degradation were evaluated by FTIR and TGA analyses. The polyurethanes showed cell viability of 53.43% and 80.37% after 1 and 10 day(s) of cytotoxicity test; and cell adhesion and proliferation in cell adhesion test. The polyurethanes produced demonstrated its potential as biomaterial for soft tissue engineering applications.

## 1. Introduction

Polyurethanes are highly demanded synthetic polymers for various industrial applications such as flexible foams for bedding and furniture, sealants, and elastomers for construction and automotive, coatings, adhesives and binder for foundry industries. Recently, biodegradable and biocompatible polyurethanes are being ventured into biomedical applications such as as polymeric materials for wound dressing [1], scaffolds for tissue engineering applications [2,3], and carriers for drug delivery systems [4]. Polyurethanes are usually produced by reacting polyhydroxyl compounds (polyester polyols or polyether polyols) with polyisocyanates in the presence of appropriate catalysts, additives, and a blowing agent. Polyols that are commonly used for the production of polyurethanes for biomedical applications are polycaprolactone (PCL) [5], polyethylene glycol (PEG) [6], polytetramethylene ether glycol [7] and hexamethylene glycol [8], in which they are all derived from the non-renewable petrochemicals. In view of the environment impact of global warming and the depletion of petroleum, research efforts in utilization of biodegradable and renewable feedstocks to substitute the petrochemical-based polyols are ongoing. Among the renewable feedstocks, vegetable oils are one of the popular resources for biodegradability, ecological friendly, and safety reasons. Besides that, vegetable oils consist of various functional sites such as ester groups and unsaturated carbon-carbon double bonds which allow them to be chemically modified to form different functional monomers. Vegetable oils, such as castor oil [9], soybean oil [10,11], rapeseed oil [12,13], canola oil [14,15] and palm oil [16,17,18,19,20,21,22,23] had been used to produce vegetable oil-based polyols for different applications of polyurethane. Castor oil with hydroxyl groups had been used to react with 1,6-hexamethylene diisocyanate to prepare the isocyanate terminated polyurethane prepolymer, in which the prepolymer was subsequently reacted with 2,3-epoxy-1-propanol (glycidol) in producing an epoxy-terminated polyurethane (EPU). The EPU was further prepared into elastomeric polyurethane films by solvent evaporation method, in the presence of 1,6-hexamethylene diamine as curing agent. However, when comparison was made to petroleum-based polyol, polyethylene glycol, the elastomeric polyurethane film prepared by polyethylene glycol was found to be more superior in hydrolytic degradation rate and mechanical properties [9]. Epoxidized soybean oil had been used as starting material to produce a vegetable oil-based polyol via a two steps synthesis method [10]. The epoxidized soybean oil was first reacted with glycerol to produce epoxidized monoglyceride. Subsequently, the epoxidized monoglyceride was reacted with lactic acid through the epoxy ring-opening reaction to produce the soybean oil-based polyol with hydroxyl value of 309.6 mg KOH/g sample and molecular weight of 480 Da. The soybean oil-based polyol was then reacted with 1,6-hexamethylene diisocyanate to produce a biocompatible polyurethane. Zieleniewska et al. reported the utilization of rapeseed oil-based polyether polyol that was derived from epoxidized rapeseed oil and diethylene glycol [12]. The rapeseed oil-based polyols were blended with the commercial polycaprolactone diol and reacted with excess amount of hexamethylene diisocyanate to produce polyurethane-urea. The polyurethane-urea was subsequently prepared into foam like scaffold (for bone tissue cultures) by using NaCl as porophore followed by annealing at 110 °C for 20 h, immersing in distilled water for 7 days to remove NaCl and drying in a vacuum dryer for 96 h at 37 °C. The scaffold underwent hydrolytic degradation, particularly at the ester linkages (backbone of the vegetable oil) of the polyurethane soft segments. The same research group also reported on employing the rapeseed oil-based polyol derived from epoxidized rapeseed oil and diethylene glycol with commercial aromatic polyester polyols (Polios 420PTA) and glycerine-based polyether polyol (Rokopol G500) with aromatic methylene diphenyl diisocyanate [13]. The polyurethane with 50% of rapeseed oil-based polyol was reported to possess satisfactory mechanical, physical and biocompatibility properties, to be used as cosmetic pumice.

Palm oil-based polyester and polyether polyols had been reported for its applications in the production of polyurethane adhesives [16], rigid and flexible foams [17,20], elastomers [20,23], coatings [22] and composites [23] aiming for various industrial applications. However, most of these polyurethanes were incorporated with either commercial petrochemical-based polyols, polyester polyol [18], polyethylene glycol (PEG) [20] and polycaprolactone diol (PCL-diol) [19,23], or petrochemical-based monomer and cross-linker, namely acrylonitrile and ethylene glycol dimethacrylate [22] in order to enhance the mechanical, thermal and biodegradation properties of the polyurethanes. For instance, Ng et al. and Pawlik and co-worker reported on the preparation of flexible polyurethanes by blending commercial petroleum-based polyols with the synthesized palm oil-based polyols. Pawlik and co-worker produced palm oil-based polyol (molecular weight of 1284 Da and hydroxyl number of 110 mg KOH/g sample) by reacting epoxidized palm oil with hexamethylene glycol [18]. The palm oil-based polyol (up to 15%) was incorporated with the commercial petrochemical-based polyether and polyester polyols to react with toluene diisocyanate in producing a flexible polyurethane. Ng et al., on the other hand, produced palm oil-based polyol (hydroxyl value = 115.72 mg KOH/g sample; molecular weight = 3270 Da) by reacting palm glycerol monostearate with glutaric acid [19]. The incorporation of 15% and 45% of the palm oil-based polyol with commercial polyols (polyethylene glycol and polycaprolactone) to react with isophorone diisocyanate produced flexible foam with enhanced mechanical properties. Reports on the utilization of vegetable oil-based polyols alone as starting material in producing biodegradable and biocompatible polyurethanes are limited.

Biodegradability and biocompatibility are the crucial aspects of biomaterials for medical and biomedical applications. Polyester elastomers prepared from biocompatible starting material such as tricarballylic acid (propane-1,2,3-tricarboxylic acid) and citric acid had been reported for its biocompatibility and biodegradation as potential scaffolds. Tricarballylic acid that generally can be derived from fumaric acid had been reacted with aliphatic diols (1,6-hexanediol, 1, 8-octanediol, 1,10-decanediol, 1,12-dodecanediol) via a polycondensation reaction to produce the prepolymer intermediate, whereby the prepolymer was further undergoing polymerization at 120 °C for 18 h to form the biodegradable and biocompatible polyester elastomer, namely poly (1,10-decanediol-co-tricarballylate) (PDET) [24]. The water absorption (water absorption = up to 60%) and mass loss of the PDET elastomers (up to 4.5% in 4 weeks) were reported to be directly proportional to the chain length of the aliphatic diols used and inversely proportional to the elastomers crosslinking density. In another work, citric acid was reacted with 1,8-octanediol via polycondensation to produce a prepolymer that was further polymerized at 60–120 °C for 1 day to 2 weeks to form the biodegradable and biocompatible poly(1,8-octanediol-co-citrate) elastomer [25]. The poly(1,8-octanediol-co-citrate) achieved ~23% of mass loss in biodegradation study, while the introduction of methyldiethanolamine (MDEA) into the cross-linking network produced poly(1,8-octanediol-co-citrate-co-MDEA) elastomer with a much improved mass loss of up to ~75%. The production of polymeric elastomers required long hour of polymerization and high temperature to complete the curing process, while for the synthesis of polyurethane-foam like material, the polymerization reaction was relatively convenient, by reacting the polyols with diisocyanate at room temperature and the polyurethanes are formed within minutes of time. As discussed, the biodegradable and biocompatible polyurethanes were ventured into biomaterial applications such as scaffold for tissue engineering [26], carriers for anti-cancer drugs [27], and biomaterials for wound dressings [28] and soft tissue adhesive [12]. Scaffold is a temporary implant which provides framework for the regeneration of new tissues and subsequently degrades and eliminates from the body gradually as waste without causing negative impact to the body [29]. Aliphatic polyester polyols are well known as biodegradable polymers since the ester linkages in the aliphatic polyester polyols are readily hydrolyzed in aqueous medium to form carboxylic acid and short chain monomers. Aliphatic polyester polyol has better biodegradability due to the aromaticity of the aromatic polyester polyol gives hydrophobic properties to the polyurethane. The degradation rate of polyurethane is also influenced by the composition, molecular structure, crystallinity and hydrophobicity or hydrophilicity of the polyurethane [30].

In view of the versatility of the polyurethanes and their prospect in medical and biomedical applications, and considering the health, safety and environmental issues that most polyurethanes in the industries are produced by the carcinogenic aromatic isocyanates and polyols from non-renewable feedstocks, we reported on the synthesis of a PPP by reacting epoxidized palm oil with malonic acid in a convenient one-pot synthesis method [31]. The previous work focused on the synthesis and optimization of the production of PPP using malonic acid, whereby the effect of reaction temperature, reaction time and functionality molar ratios (epoxy: carboxyl) of EPO and malonic acid on the physico-chemical properties of the polyols were discussed. The objective of the present work is to discuss the production of a biodegradable polyurethane biomaterial by PPP that was derived by a longer carbon chain length of dicarboxylic acid (glutaric acid) and aliphatic isophorone diisocyanate. The focus of the present work is to discuss the effects of water content and isocyanate index used in the polyurethane formulation on the physical, chemical, mechanical, and enzymatic degradation properties of the polyurethanes. In addition to the biodegradation properties assessed in the present work, the polyurethane produced by PPP derived by glutaric acid was made in comparison with the previous work, which reported on polyurethane produced by PPP derived by malonic acid. The biocompatibility potential of the polyurethanes was also being investigated through MTT assay and cell adhesion study.

## 2. Materials and Methods

### 2.1. Materials

Epoxidized palm olein (EPO) (OOC = 3.5%, acid value = 0.77 mg KOH/g sample) was procured from Advanced Oleochemical Technology Division of Malaysian Palm Oil Board (MPOB)(Bandar Baru Bangi, Malaysia). Glutaric acid, glycerol 99%, dibutyltin dilaurate (DBTL) and isophorone diisocyanate (IPDI) were purchased from Merck. Niax A33, triethylene diamine and Niax L580 silicone surfactant were purchased from SamChem Holding Sdn. Bhd. Polyethylene glycol (PEG, number average molar mass, Mn = 6000 g/mol) and polycaprolactone diol (PCL diol, Mn = 2000 g/mol) were purchased from Fischer Scientific (MA, USA). Porcine pancreas type II lipase (100–400 U/mg protein) and thiozolyl blue tetrazolium bromide (MTT) were purchased from Sigma-Aldrich (MO, USA). Dulbecco’s modified eagle medium (DMEM), fetal bovine serum (FBS) and penicillin-streptomycin (Brand: Gibco) were purchased from Bio-diagnostics (Giza, Egypt). Latex urinary (Idealcare^®^, Kedah, Malaysia) and feeding (Ryle’s tube) catheters were purchased from a local pharmacy outlet. Human osteosarcoma MG63 cells were obtained from the cell bank of International Medical University (IMU, Kuala Lumpur, Malaysia). All chemicals and reagents used in the analyses were of analytical grade.

### 2.2. Synthesis of Palm Oil-based Polyester Polyols (PPP)

Epoxidized palm olein (EPO) was reacted with glutaric acid at the optimized functionality molar ratio (epoxy: carboxyl) of 1:0.7 and reaction temperature of 210 °C for 6 h to produce PPP. The reaction was carried out in a three-neck round bottomed flask equipped with a thermometer, condenser, and a magnetic stirrer. Oxirane oxygen content, acid value and OHV of PPP were conducted according to AOCS official method Cd 9-57 oxirane oxygen content, AOCS official method Te 2a-64 acid value and AOCS official method CD 13-60 hydroxyl value, respectively. The number average molecular weight (M_n_) of the PPP was analyzed by gel permeation chromatography (GPC) using Viscotek GPCmax VE 2001 (Malvern Panalytical, Cambridge, United Kingdom). The viscosity of PPP was measured by DHR-2 rheometer (TA Instruments, New Castle, DE, USA) at 25 °C and 40 °C, while the cloud point and pour point were conducted according to ASTM D 97-06, standard test method for pour point of petroleum products and AOCS official method CC 6-25 cloud point test, respectively. The physico-chemical properties of PPP are summarized in Table 1.

### 2.3. Production of Polyurethane

Polyurethane was produced by one-shot foaming method. PPP, A33 amine catalyst, L580 silicon surfactant, DBTL gelling catalyst and water blowing agent were mixed homogenously at 1300 rpm using an IKA EUROSTAR Digital mechanical stirrer (Staufen, Germany) for 2 min. Then, IPDI was added into the reaction mixture and stirred for another 3 min. The reaction mixture was poured into a polyethylene container and allowed to rise freely at room temperature and cure for 24 h. The production of polyurethane was conducted based on the standard formulation (Table 2) and the optimum polyurethane formulation was determined by the alteration of water content ranged from 0.6–1.4 g and isocyanate index ranged from 0.8–1.2. The physical, chemical, mechanical, and enzymatic degradation properties of the polyurethanes were evaluated. As comparison for biocompatibility study, a reference polyurethane (Pu-ref) was produced by commercial polyols, PEG 6000, PCL diol 2000 and IPDI at isocyanate index 1.0. The optimal formulation of PU-ref is shown in Table 2.

### 2.4. Characterization of Polyurethane

#### 2.4.1. Morphology, Density and Porosity of Polyurethane

The pore size and cell distribution of polyurethane were determined using Hitachi TM3000 scanning electron microscope (Hitachi High-Tech, Tokyo, Japan). The sample was coated with gold using a vacuum sputter coater. The core density was determined by measuring the mass and volume according to the ASTM D3574-Test A, standard test methods for flexible cellular materials, 2011. Samples were cut into cubic shapes (1 cm × 1 cm × 1 cm) and the core density and core porosity (εC) were calculated according to the following equations:

(1) $$\mathrm{Density}=\frac{\mathrm{Mass}\;\left(\mathrm{kg}\right)}{\mathrm{Volume}\;\left({\mathrm{cm}}^{3}\right)}$$

(2) $$\varepsilon C=1-\left(\frac{\rho C}{\rho P}\right)\frac{\rho P-\left(\frac{\rho A\rho P}{\rho c}\right)}{\rho P-\rho A}$$

where ρC = density of sample, ρP = specific gravity of polyurethane, 1200 kg m^−3^, ρA = specific gravity of air, 1.29 kg m^−3^

#### 2.4.2. ATR-FTIR Analysis

The functional groups of the polyurethane were determined using Perkin-Elmer Spectrum 400 (Perkin Elmer, MA, USA) with 4 scans for each run in the range of 450 cm^−1^ to 4000 cm^−1^. The polyurethanes were placed onto the diamond crystal and subjected to FTIR scanning. The changes in the chemical structure of selected polyurethanes before and after enzymatic degradation were also evaluated.

#### 2.4.3. Thermal Analysis

Thermal analysis of the samples was performed using Perkin Elmer simultaneous thermal analyzer (STA) coupled with TGA 4000 (Perkin Elmer, MA, USA). The samples were analyzed at temperature ranged from 30 to 800 °C at the heating rate of 10 °C/min under nitrogen atmosphere.

#### 2.4.4. Mechanical Properties

The tensile test and compression test were measured using a Universal Instron machine (Instron, MA, USA) according to the ASTM D 3574-Test E, standard test methods for flexible cellular materials and DIN 53577, determination of compression stress value and compression-strain characteristics flexible cellular materials. Tensile test was performed on the pre-cut dumbbell shaped polyurethane under the crosshead speed of 500 mm/min. The tensile strength (kPa), elastic modulus, and elongation at break (%) were determined. Compression test was conducted on the pre-cut rectangular shaped polyurethane (5 cm × 5 cm × 2.5 cm). The sample was compressed to 70% of its original height under 100 mm/min and 40% of the compression stress (kPa) was determined. All analyses were performed in at least three replicates and mean ± SD of the readings were recorded.

#### 2.4.5. In Vitro Enzymatic Degradation

Polyurethane samples were cut into 6 mm in diameter and the mass of the polyurethanes were in the range of 10–15 mg [32]. The specimens were immersed in 3 mL of phosphate buffered solution (PBS) (pH 7.4) containing 1 mg/mL of porcine pancreas lipase [33]. The sample was incubated in a 37 °C shaking water bath at 30 rpm for 28 days. The enzymatic PBS was replenished daily to maintain the enzyme activity. At each time point, the polyurethane specimens were removed from the solution and any dripping water was removed using absorbent paper before measuring for its mass. The polyurethanes were then dried at 50 °C and the dry mass was measured. The degradation products were also subjected to TGA and FTIR analyses.

(3) $$\mathrm{Water}\;\mathrm{absorption}=\frac{\mathrm{Wet}\;\mathrm{weight}-\mathrm{Initial}\;\mathrm{weight}}{\mathrm{Initial}\;\mathrm{weight}}\times 100\%\;(\mathrm{Skrobot}\;\mathrm{et}\;\mathrm{al}.,\;2015)$$

(4) $$\mathrm{Mass}\;\mathrm{loss},\;\%=\frac{\mathrm{Initial}\;\mathrm{weight}-\mathrm{Final}\;\mathrm{weight}}{\mathrm{Initial}\;\mathrm{weight}}\times 100\%\;(\mathrm{Skrobot}\;\mathrm{et}\;\mathrm{al}.,\;2015)$$

#### 2.4.6. Cytotoxicity Test

Sample Preparation

The cytotoxicity test was conducted by the extract method [34] whereby the medium used in incubating the polyurethane samples was used for MTT assay. Polyurethane samples were first cut into 5 × 5 × 4 mm^3^ rectangular shape and sterilized using ultraviolet in a biosafety cabinet for 30 min [35]. After sterilization, the polyurethane samples were immersed into 2 mL of DMEM containing 10% fetal bovine serum (FBS) and 1% penicillin/ streptomycin incubated at 37 °C in a CO_2_ incubator for 1 and 10 days, respectively. The medium was collected after 1 day and 10 days of incubation for the subsequent MTT assay.

MTT Assay

MG-63 human osteosarcoma bone fibroblasts at a cell density of 1 × 10^4^ cells/well (100 μL) were seeded into 96-well plates and incubated for 24 h. After 24 h, the medium was removed and replaced with the extract medium collected after 1 day and 10 days of incubation. The 96-well plates were then incubated for another 24 h. After that, 20 μL of the extract medium was replaced with 20 μL of 3-(4,5-dimethylthiazol-2-yl)-2,5-diphenyltetrazolium bromide (MTT) solution and incubated for 4 h. After 4 h of incubation, the extract medium was removed and 100 μL dimethyl sulfoxide (DMSO) was added into each well. The absorbance of the solution was measured using a microplate reader (Tecan, Männedorf, Switzerland) at the wavelength of 570 nm. The cell viability was calculated using the following formula:

(5) $$\%\;\mathrm{Cell}\;\mathrm{Viability}=\frac{\mathrm{Absorbance}\;\mathrm{of}\;\mathrm{extracted}\;\mathrm{medium}\;\mathrm{with}\;\mathrm{cells}-\mathrm{Absorbance}\;\mathrm{of}\;\mathrm{extracted}\;\mathrm{medium}}{\mathrm{Absorbance}\;\mathrm{of}\;\mathrm{cells}\;\mathrm{with}\;\mathrm{medium}-\mathrm{Absorbance}\;\mathrm{of}\;\mathrm{medium}}$$

#### 2.4.7. Cell Adhesion Test

Cell adhesion was used to study the attachment of the cells onto the surface of the polyurethane. The polyurethanes were cut into cube form with a diameter of 10 mm and a thickness of 4 mm. The samples were sterilized by ultraviolet light for 30 min on each side of the samples for disinfection, followed by washing with sterile PBS, and incubated in DMEM at 37 °C for 24 h. After 24 h of immersion, the polyurethane samples were removed and placed into a new 24-well plate. MG-63 cells (100 μL) at a cell density 5 × 10^4^ cells/well were seeded onto the polyurethane samples and placed into a CO_2_ incubator at 37 °C for 1 h for cell attachment. After that, 500 μL of DMEM containing 10% FBS and 1% penicillin/streptomycin were added into each well. The polyurethane samples were incubated for another 12 h and 24 h. At each time point (12 and 24 h), the samples were removed from DMEM and rinsed gently with PBS to remove unattached cells. The attached cells were then fixed with 3% paraformaldehyde for 30 min. Then, the sample was rinsed with PBS for 5 min, followed by the dehydration of the samples in a serial concentration of ethanol solutions (30, 40, 50, 70, 90, 95 and 100%) for 10 min each. The polyurethane samples were dried overnight before being visualized under an SEM (Hitachi TM3000, Tokyo, Japan) to record the morphology of the cells adhered onto the polyurethane samples.

## 3. Results and Discussion

### 3.1. Production of Biodegradable Polyurethane

**Figure 1:**
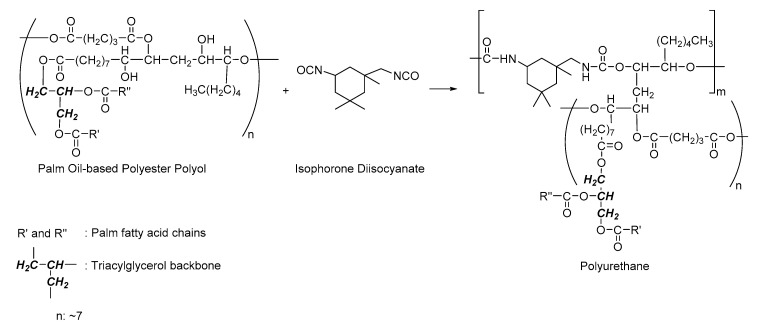
Reaction scheme of palm oil-based polyester polyol reacted with IPDI in producing polyurethane.

The newly synthesized PPP with molecular weight of 6698 Da and OHV of 84.5 mg KOH/g sample (Table 1) was used to react with aliphatic isocyanate IPDI to produce a biodegradable flexible polyurethane (Figure 1). Polyols with high molecular weight ranged from 2000–10,000 Da and a smaller number of hydroxyl groups are meant for flexible polyurethane production, while polyols with low molecular weight and high number of hydroxyl groups are intended for rigid polyurethanes [36]. The aim of this work was to produce biodegradable and biocompatible polyurethane for soft tissue engineering; hence the newly synthesized PPP was an ideal candidate. Aliphatic isocyanate has been known for its lower reactivity than that of aromatic isocyanate, hence the polyurethane industry was mainly producing polyurethane by employing aromatic isocyanate, namely toluene diisocyanate (TDI) and diphenylmethane diisocyanate (MDI) [37]. However, polyurethanes produced from aromatic isocyanates known for its drawback of toxic degradation compounds such as aromatic amines [38] which are carcinogenic and can cause adverse effects in large enough quantity. For instance, MDI-derived polyurethanes had been reported on producing a carcinogenic degradation product, methylene diamine (MDA) [39], hence limiting its applications in medical and biomedical applications. In this work, PPP was used to react with IPDI without the incorporation of other petrochemical-based polyols, and a one-shot foaming method was employed in the polyurethane production. One-shot foaming method is known as the most convenient, cost effective and environmentally friendly method without involvement of any solvent medium and external heating energy. It provides an efficient mixing of all the materials in a single step and react at room temperature. Water was used as blowing agent in polyurethane production by reacting water with IPDI in forming amine and carbon dioxide. The effects of water content and isocyanate index on the polyurethanes produced were studied.

### 3.2. The Effect of Water Content

**Figure 2:**
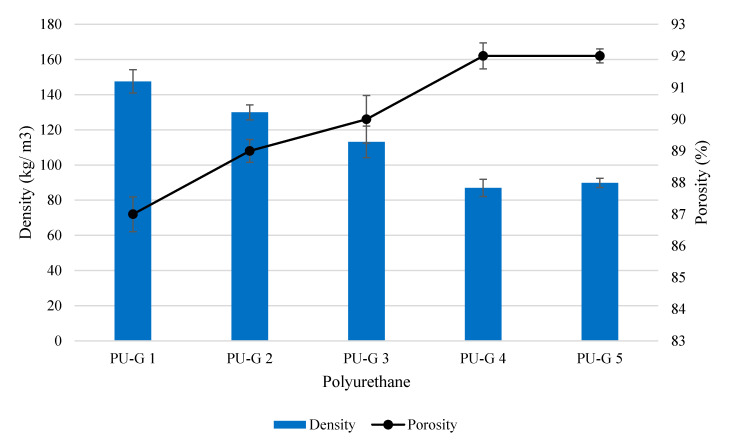
Density and porosity of polyurethanes produced by different water content of 0.6 g (PU-G 1), 0.8 g (PU-G 2), 1.0 g (PU-G 3), 1.2 g (PU-G 4) and 1.4 g (PU-G 5) (Mean ± SD, *n* = 3).

The polyurethanes were first prepared by using different water contents (0.6, 0.8, 1.0, 1.2 and 1.4 g) while the amounts of all other additives and isocyanate index (1.0) were fixed (Table 2). The polyurethanes produced were denoted as PU-G 1 to 5. Overall, the density and porosity of the polyurethanes PU-G 1 to 5 were in the range of 87–147 kg/m^3^ and 87–92% (Figure 2). The increased water content showed a decremental trend in the density and incremental trend in the porosity of the polyurethanes. This was due to the increased water content in the polyurethane formulations causing the increased production of urea and carbon dioxide. The carbon dioxide provided volume for the expansion of bubbles to form open pores in the polyurethane [40]. Hence, the densities of the polyurethanes PU-G 4 (density = 87.01 kg/m^3^) and PU-G 5 (density = 89.86 kg/m^3^) produced by higher water content of 1.2 g and 1.4 g were lower as compared to PU-G 1 (density = 147.53 kg/m^3^) produced by only 0.6 g of water. Nevertheless, excessive water used in the production of polyurethane may cause negative pressure and cell deformation because of the rapid diffusion of carbon dioxide through the cell wall of the polyurethane [41].

**Figure 3:**
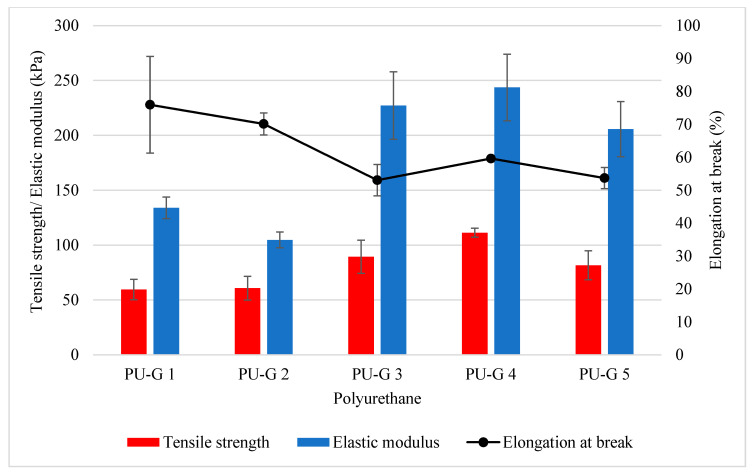
Tensile strength, elastic modulus and elongation at break of polyurethanes produced by different water content of 0.6 g (PU-G 1), 0.8 g (PU-G 2), 1.0 g (PU-G 3), 1.2 g (PU-G 4) and 1.4 g (PU-G 5) (Mean ± SD, *n* = 3).

For the tensile properties of the polyurethanes, the increased amount of water from 0.6 g to 1.2 g used in the formulation had produced polyurethanes with increased tensile strength and elastic modulus, wherein the tensile strength and elastic modulus of PU-G 1 were increased from 59.51 kPa and 133.97 kPa to 111.25 kPa and 243.69 kPa, respectively (Figure 3). However, when higher water content of 1.4 g (PU-G 5) was used in the formulation, the tensile strength and elastic modulus of PU-G 5 were reduced to 81.56 kPa and 205.72 kPa. A higher amount of water produced more urea contents when water reacts with isocyanate. The urea hard segment enhanced the mechanical properties of the polyurethanes as reflected by the enhanced tensile strength and elastic modulus. Nonetheless, the surplus of urea contents could cause brittleness in the polyurethane and is thus reflected in lower value of tensile strength and elastic modulus in PU-G 5 (1.4 g of water). As for the elongation at the break of the polyurethanes, generally, polyurethanes produced by higher water content were experiencing a detrimental effect from 75.98% (PU-G 1) to 53.7% (PU-G 5) (Figure 3). The elongation at break was basically inversely correlated with the tensile strength, due to polyurethanes with higher tensile strength being less deformable materials that break at lower strain. This observation was in agreement with the work reported by Dworakowska et al. where the elongation at break of the polyurethanes produced by blending of rapeseed oil-based polyols with petroleum-based polyether polyol were decreased to ~50% (from ~105%) when the polyurethane achieved higher tensile strength of ~90 KPa (from ~75 kPa) [42]. Considering formulation PU-G 4 (1.2 g of water content) produced polyurethanes with the highest tensile strength of 111.25 kPa and elastic modulus of 243.69 kPa, and an acceptable elongation at break of 59.63%, the water content of 1.2 g was selected to further optimize the formulation of the polyurethanes by evaluating the effect of isocyanate index on the physical, chemical, mechanical, and thermal, as well as biodegradation, properties of the polyurethanes.

### 3.3. The Effect of Isocyanate Index

The polyurethanes were further optimized by altering the isocyanate index ranged from 0.8 to 1.2 and the polyurethanes were denoted as PU 0.8, PU 0.9, PU 1.0, PU 1.1 and PU 1.2, respectively. Generally, the density and porosity of polyurethanes did not show a significant relevant trend associated to the isocyanate index used in the formulation (Table 3). The density of the polyurethanes was in the range of 70–96 kg/m^3^ and all the polyurethanes were recorded with high porosity of 92–94%. Ryszkowska, Auguscik, Sheikh and Baccaccini reported that polyurethanes with porosity of more than 70% was adequately applied in bone tissue engineering [43]. Polo-Corrales and co-workers have also reported that scaffolds with 75–90% porosity permit ingrowth of cancellous bone tissue and scaffolds with > 90% porosity is used in many scaffold designs due to high porosity scaffolds allow adequate diffusion of nutrients during tissue culture and provides sufficient surface area for cell-biomaterial interactions [40].

**Figure 4:**
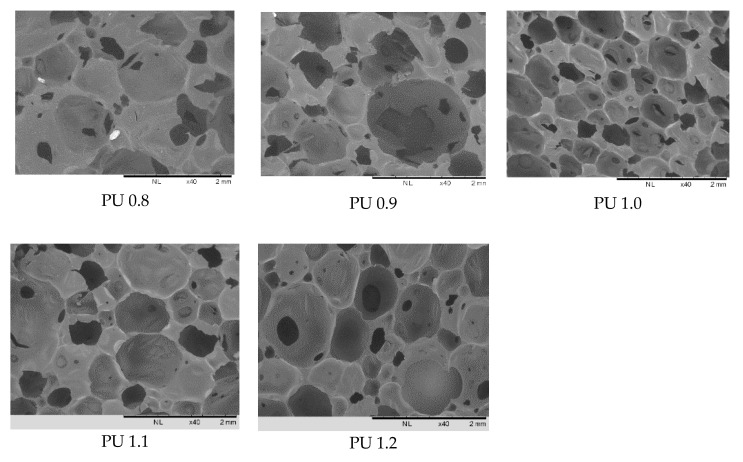
SEM images of polyurethanes produced by different isocyanate indices (40× magnification) PU 0.8, PU 0.9, PU 1.0, PU 1.1, PU 1.2 were prepared by reacting palm oil-based polyester polyol with IPDI at isocyanate index of 0.8, 0.9, 1.0, 1.1 and 1.2.

On the other hand, the increased isocyanate index in the formulation had caused an increment of pore size from 37–1333 µm (PU 0.8) to 57–1700 µm (PU 1.2), respectively (Table 3). The increased isocyanate index in the polyurethane formulation from 0.8 to 1.2 had increased the pore sizes of the polyurethanes produced due to the combination of two types of conflict forces effect [44]. Higher amounts of IPDI used in the reaction could lead to the formation of allophanate crosslinking and hence increased the elasticity of the pore channels. This phenomenon could cause difficulty in growing and coalescence of the foam bubbles. The morphology and pore size of the biomaterial used as scaffold are important aspects for cell proliferation and regeneration of new tissue. Hence, the cross-sectional morphology of the polyurethanes produced by different isocyanate indices was examined by SEM (Figure 4). All the polyurethanes showed spherical and interconnected micropores and macropores (more than 75 μm) with the pore size ranged of 37–1700 μm (Table 3). Interconnected pores of the polyurethane scaffolds are crucial to ensure correct cell attachment, proliferation, and tissue ingrowths [45]. It has been reported that polyurethanes with interconnected pores and pore sizes of 150–355 μm allowed meniscal reconstruction [46], polyurethanes with 65–426 μm pore size had the potential as scaffold for cardiovascular and soft tissue engineering applications [47], interconnected polyurethane with pore size of up to 5000 μm allowed the penetration of MC 3T3 cells for bone and soft tissue engineering applications [48]. In addition, an ideal biomaterial for tissue engineering scaffolds should exhibit high porous foam-like with macroporous and microporous structure to allow continuous flow of nutrients in the scaffold and be populated with cells of various origins [49]. The polyurethanes PU 0.8–PU 1.2 produced have shown their potential application as biomaterials for scaffolds in various tissue engineering applications due to their porosity, pore size, and macroporous and microporous as well as interconnected structure for cell growth and tissue regeneration (Table 3 and Figure 4).

In terms of mechanical properties, the increasing isocyanate index from 0.8 to 1.0 had increased the tensile strength of the polyurethanes from 48 kPa to 111 kPa (Table 3). However, the tensile strength was reduced to 70–85 kPa when the formulation was further altered with a higher isocyanate index of 1.1 and 1.2. This was mainly due to the more complete reaction of isocyanate with PPP in PU 1.0 as compared to PU 0.8 and PU 0.9 in producing urethane hard segment, hence increased the tensile strength with lower elongation at break. However, beyond a certain yield point (isocyanate index > 1.0), the polyurethane exhibited strain hardening and caused the earlier break at maximum strain as shown in the lowering of tensile strength [50]. On the other hand, it was observed that the elongation at break was in inverse-relationship with the increasing isocyanate index while the elastic modulus was directly proportional with the increasing isocyanate index since materials with higher elastic modulus indicating stiffness and lower flexibility. The elongation at break of the polyurethanes was decreased from 76.62% to 32.12%, whereas the elastic modulus was increased from 79.83 kPa to 482.69 kPa when higher isocyanate index from 0.8 to 1.0 was used in the polyurethanes production. The tensile behavior of the polyurethane is mainly dependent on the concentration of the hard and soft segments and the intermolecular hydrogen bonding between the hard and soft segments that making the polyurethane becoming more rigid [51]. The decreased in elongation at break at higher isocyanate index could be due to the increased amounts of urethanes, urea and hydrogen bonded urea in the polymer matrices which had limited the motion of the polyurethane chain [52].

On the other hand, the compression stress of the polyurethanes exhibited significant enhancement from 15.10 kPa to 115.09 kPa along with the increment of isocyanate index used in the polyurethane production from 0.8 to 1.2 (Table 3). Higher density polyurethanes with less porous structures are known to exhibit higher compression stress. Similar to the report by Prociak et al. (2016) [53], the compression stress of the polyurethanes in this work did not show a relevant relationship with the density of the polyurethanes (Table 3). This can be explained by the fact that the mechanical strength was not only influenced by the characteristics of the porous structure but in this case, it was more prominently affected by the hard segment contents in the polyurethanes [54]. A higher amount of isocyanates could result in more cross-linking between the reaction of isocyanate and the hydroxyl groups of the polyols, hence reflected in improved compression stress [55]. Besides, the increment of compression stress may be attributed to the increase in intermolecular forces in the hard segments of the polyurethanes when higher amount of isocyanates were used in the reaction [56]. The excess of isocyanates could also potentially produce higher amount of isocyanurates [57], urea and biuret [58]—hard segments in the polymer matrices that subsequently increased the compression stress—since these hard segments cause a stiff structure in polyurethanes.

Gorna et al. reported that polyurethane with the compression stress range 20–400 kPa was applicable for bone graft substitutes [58], while Hafeman et al. reported the biodegradable polyurethane scaffolds produced by PCL trifunctional polyester polyols and hexamethylene diisocyanate trimer or lysine triisocyanate with a compression stress range 5–85 kPa were potential biomaterials for bone and soft tissue engineering [47]. Besides, Gogolewski et al. reported on a poly(ethylene oxide) and PCL-based polyurethane scaffold with compression stress ranging from 17 to 230 kPa provided the ingrowth of new bone in sheep iliac crest [42]. In this work, the newly synthesized polyurethane produced by 100% PPP and IPDI at a different isocyanate index had demonstrated its prospect as biomaterial for tissue engineering scaffolds, particularly in tissue repair and regeneration.

### 3.4. ATR-FTIR Spectroscopy

**Figure 5:**
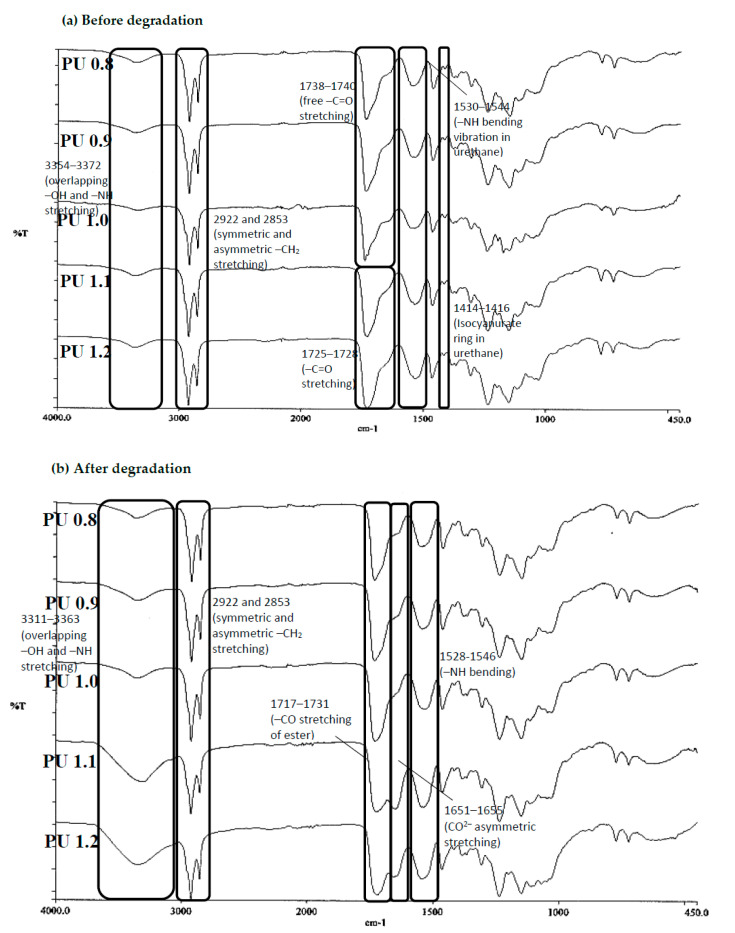
FTIR spectra of polyurethanes produced by different isocyanate indices (**a**) before and (**b**) after enzymatic degradation study. PU 0.8, PU 0.9, PU 1.0, PU 1.1, PU 1.2 were prepared by reacting palm oil-based polyester polyol with IPDI at isocyanate index of 0.8, 0.9, 1.0, 1.1 and 1.2.

ATR-FTIR was used to determine the chemical groups, the extent of hydrogen bonding and interaction between the hard and soft segments of the polyurethanes (Figure 5a). The characteristic peaks of overlapping of hydrogen bonded –NH and –OH stretching vibration (3354–3372 cm^−1^), symmetric and asymmetric methylene stretching vibration (2922 cm^−1^ and 2853 cm^−1^), –C=O stretching vibration (1725–1728 cm^−1^), –NH bending vibration (1530–1544 cm^−1^) for the urethane and –C–O stretching vibration (1031–1064 cm^−1^) of acid and alcohols were observed in all the FTIR spectra of the polyurethanes produced by different isocyanate indices, indicating the reaction of the PPP with IPDI. The absence of free –NCO stretching (2273 cm^−1^) in all polyurethanes including PU 1.1 and PU 1.2 produced with higher isocyanate index evident the completion of the reaction [59]. However, there was a small and low peak intensity at the wavelength of 1414–1416 cm^−1^ indicating a small amount of isocyanurate ring structures observed in all the polyurethanes. An isocyanurate ring is produced through the cyclotrimerization of the isocyanate in the polyurethane structure, which could potentially improve the mechanical strength of polyurethane.

The intensity and the position of the –NH stretching and –C=O stretching vibrations were also used to evaluate the phase separation of polyurethane [60]. The degree of microphase separation of polyurethane is dependent on the extent of hydrogen bonding in the hard segment [51]. The –NH of urea functional groups and carbonyl oxygen in urethanes were the main attribution of the intermolecular forces such as hydrogen bonding between the hard segments. Besides that, the –NH of urea may participate in hydrogen bonding with the ester oxygen in hard and soft segments of polyurethane [51]. The FTIR spectra of all the polyurethane samples were observed to have a small and broad peak at the wavelength of 3354–3372cm^−1^ indicated weak –NH hydrogen bonding of the urea groups. Besides, broad and free –C=O stretch of the urethane detected at 1738–1740 cm^−1^ in FTIR spectra of PU 0.8–PU 1.0 was found to be shifted to 1725–1728 cm^−1^ to a lower frequency (red shift) in spectra of PU 1.1 and 1.2 (1725–1728 cm^−1^). This demonstrated the increment of –C=O hydrogen bonding stretching in urethane when a higher amount of IPDI was used in the polyurethane production. The increase in hydrogen bonding has the potential to increase the degree of microphase separation which will in turn improve the mechanical properties of the polymer [61]. The FTIR observation agreed with the assumption made that higher IPDI enhanced the compression stress of the polyurethanes due to the increase in intermolecular forces in the hard segments of the polyurethanes.

### 3.5. Thermal Analysis

**Figure 6:**
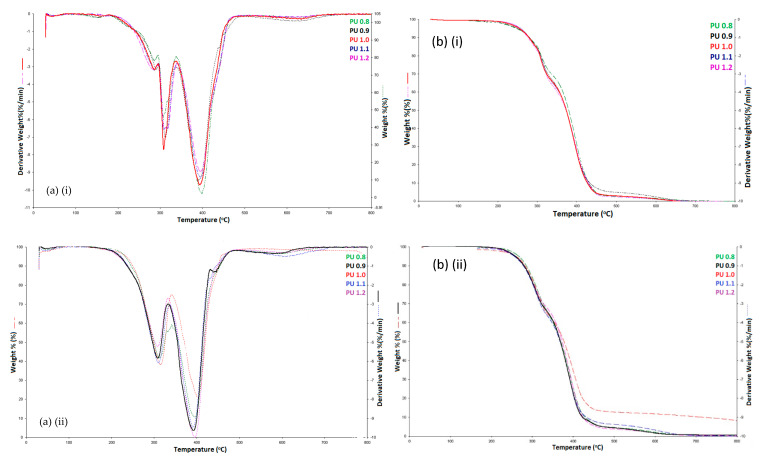
(**a**) DTG curves of polyurethanes produced by different isocyanate indices (**i**) Before degradation (**a**) (**ii**) After degradation and (**b**) TG curves of polyurethanes produced by different isocyanate indices (**i**) Before degradation (**b**) (**ii**) After degradation.

Thermogravimetric analysis (TGA) was used to study the thermal stability of the polyurethanes produced by different isocyanate indices through the analysis of the mass change (TG) and the derivative of the mass change curve (DTG) of the polymer (Figure 6). The 5% decomposition of polyurethane (T_5%_) started at temperatures ranging from 248 to 258 °C and ended at temperatures ranging from 646 to 780 °C (Table 4). Three stages of the decomposition process were observed in the polyurethane samples. The first stage of decomposition showed the loss of volatile compounds such as additives (catalyst and surfactant) used in the production of polyurethanes [62] at the first maximum degradation temperature (T_max1_) ranged from 281 to 287 °C with mass loss of 12–13%. The second stage of decomposition at the second maximum degradation temperature (T_max2_) ranged from 306 to 309 °C and the third stage of decomposition exhibited by the third maximum degradation temperature (T_max3_) ranged from 394 to 398 °C. The second stage of polyurethane decomposition corresponded to the dissociation of urethane linkages to form isocyanate and alcohol, primary amine and olefin, secondary amine and carbon dioxide [49]. Meanwhile, the third stage of polyurethane decomposition involved the rupture of ester linkages and fatty acid chains of the polyurethane soft segments [63]. The decomposition temperature of ester linkages was higher than that of the urethane linkages due to the high thermal stability of esters. The polyurethanes produced by isocyanate indices ranging from 0.8 to 1.2 had shown full decomposition at 800 °C without any residue left. High thermal temperature was needed to fully decompose the polyurethane samples due to the presence of small amounts of isocyanurate hard segments (as evident in the FTIR analysis) with high degradation temperatures ranging from 400–800 °C. Besides that, there was slight decrement in the decomposition temperature of polyurethane at 50% mass loss (T_50%_) from 379 to 372 °C for polyurethanes prepared with the increase in isocyanate index from 0.8–1.2. This was probably due to the increase in urethane linkages that involved in decomposition at T_max1_ [64]. Referring to a previous work reported on PCL reacted with excess amount of 1,6-hexamethylene diisocyanate (HDI) (molar ratio of PCL: HDI = 1:2) for the production of biodegradable and biocompatible elastomeric polyurethanes meant for bioengineering applications [64], the degradation temperature of the hard segment of the elastomeric polyurethanes were ranged from 211–229 °C while the degradation temperature of the soft segments were ranged from 343–374 °C [35]. Meanwhile, in the present work, the degradation temperature of the hard segment (T_max2_) of the palm oil-based polyurethane foam-like material were ranged from 306–309 °C and the degradation temperature of the soft segment (T_max3_) were ranged from 394–398 °C (Table 4). When referring to polyurethanes prepared by other vegetable oil-based polyols, Prociak et al. reported that when 50% of rapeseed oil-based polyol was incorporated into the petrochemical-based polyether polyol in the preparation of polyurethane, the T_50%_ of the polyurethane was increased from 306 °C to 343 °C. The authors also reported that the T_50%_ of the polyurethane prepared by incorporation of 30% of palm oil-based polyol with petrochemical-based polyether polyol had been enhanced from 297 °C to 326 °C [65]. This shows that polyurethanes derived from vegetable oil-based polyols enhanced the thermal properties of the polyurethanes in some instances.

### 3.6. In Vitro Enzymatic Degradation

The polyurethanes prepared by different isocyanate indices (PU 0.8–PU 1.2) were evaluated for their biodegradability via in vitro enzymatic degradation. Porcine pancreas type II lipase was used in the in vitro degradation process to simulate the enzymatic biodegradation process in human body. The water uptake and mass loss of the polyurethanes, as well as pH changes in the medium, and compression stress after the enzymatic degradation treatment were evaluated for 28 days of study. In addition, the chemical, thermal and morphology changes of the polyurethanes were studied by ATR-FTIR, TGA and SEM analyses.

#### 3.6.1. Water Uptake

**Figure 7:**
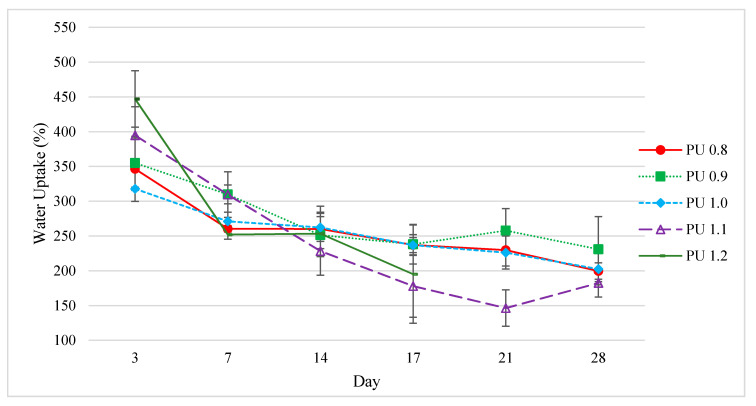
Water uptake of polyurethanes produced by PPP using different isocyanate indices after enzymatic degradation test (Mean ± SD, *n* = 3). PU 0.8, PU 0.9, PU 1.0, PU 1.1, PU 1.2 were prepared by reacting palm oil-based polyester polyol with IPDI at isocyanate index of 0.8, 0.9, 1.0, 1.1 and 1.2, respectively.

Water absorption is an important parameter to determine the degradation of polyurethane as it is the first stage of biodegradation process. All the polyurethanes showed extremely high-water absorption with 300–450% water uptake after 3 days of incubation, and 180–230% water uptake after 28 days of incubation (Figure 7). The high water uptake of all the polyurethanes could be attributed to the high porosity (92–94%) of the polyurethanes. High water absorption is a beneficial characteristic for polymers aimed at biomedical applications, since the wettability of the polymer materials has the advantage of compatibility with the internal environment of the body with high water content [66]. Polyurethane scaffolds modified with clay nanoplates, which were designed for bone tissue engineering, had been reported as demonstrating water uptakes of 27% and 38% after 16 h of immersion [67]. In another study, biodegradable polyurethane microspheres synthesized from hydroxyethyl methacrylate, poly(hexamethylene carbonate) and lysine diisocyanate meant for biomacromolecule delivery and tissue regeneration applications were reported to have a water uptake of 98% after 3 days of immersion [68]. Moreover, with a biodegradable polyurethane synthesized from PEG and L-lactide and hexamethylene diisocyanate, the water uptake of the polyurethane scaffold was 229.7% after 10 h of water immersion [32]. The PEG and L-lactide-derived polyurethane scaffold was tested for in vitro cytocompatibility and in vivo biocompatibility for hypopharyngeal tissue engineering purposes.

The water absorption was as expected decreased along with the mass reduction in the polyurethanes when the biodegradation period prolonged (Figure 7). The trend of water uptake characteristic for the tested polyurethane samples was found to correspond to the pore size (Table 3) of the polyurethanes, i.e., PU 1.2 > PU 1.1 > PU 0.9 > PU 0.8 > PU 1.0. The large pore size and high porosity of the polyurethanes allowed large amount of enzymatic solution to be penetrate and be absorbed into the polyurethanes.

#### 3.6.2. Mass Loss

**Figure 8:**
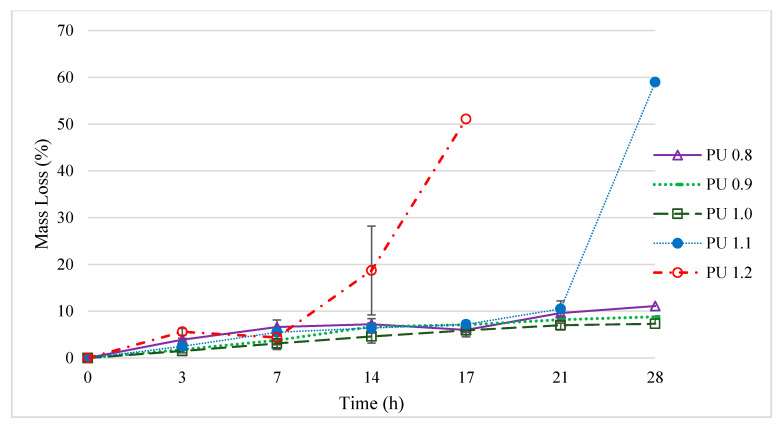
Mass loss of polyurethanes produced by PPP using different isocyanate indices after enzymatic degradation test (Mean ± SD, *n* = 3). PU 0.8, PU 0.9, PU 1.0, PU 1.1, PU 1.2 were prepared by reacting palm oil-based polyester polyol with IPDI at isocyanate index of 0.8, 0.9, 1.0, 1.1 and 1.2, respectively.

**Figure 9:**
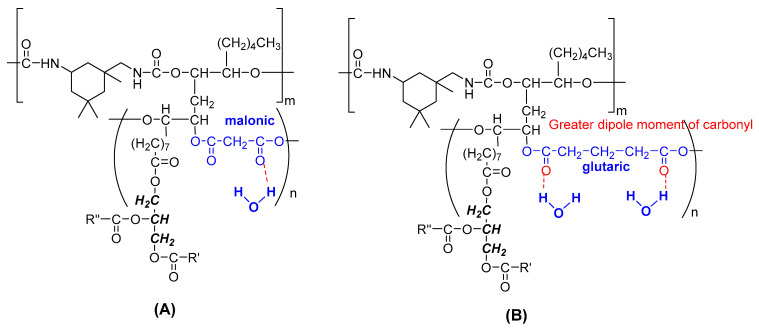
Hypothesised hydrogen bonding formed between ester groups of the (**A**) malonic acid moiety (**B**) glutaric acid in the soft segment of the polyurethanes with water molecules during the enzymatic degradation study.

The second stage of the degradation is known as the induction stage, in which ester linkages in the polymer are hydrolyzed, and the third stage of degradation is the erosion stage, which causes the mass loss of the polymer [35]. The mass loss of all polyurethanes prepared (PU 0.8–PU 1.2) remained almost constant for the first week of degradation due to the washing of the uncross-linked top layer of the surface of the polymer (Figure 8) [69]. The degradation rate was more significant after 14 days of incubation, in which the mass loss of the polyurethanes was corresponded to the water uptake characteristic of the polyurethanes, where PU 1.2 and PU 1.1 recorded the highest mass loss of 51% and 59% after 17 days and 28 days of enzymatic incubation, respectively. PU 1.2 exhibited the fastest degradation rate (51.1% of mass loss) after 17 days of incubation, and was not measurable after day 17 due to fragmentation of the polyurethane. On the other hand, PU 1.0, PU 0.9 and PU 0.8, with lower water uptakes of 199–231%, were relatively stable; demonstrating a mass loss of 7.3%, 8.8% and 11.1%, respectively, for 28 days of incubation. A comparison was made with our previous report on the polyurethane prepared with malonic acid derived palm oil-based polyol [31], whereby the biodegradation rate of the present work has shown much improvement in terms of mass loss and water uptake. The previous work reported that the water uptake of the polyurethanes was only up to 145% and the maximum mass loss was 15.3% for 28 days of enzymatic incubation, when malonic acid was used as reactant in the palm oil-based polyol synthesis. This shows that dicarboxylic acid plays a crucial role in the soft segment moiety of the polyurethanes. Glutaric acid [(CH_2_)n(COOH)_2,_ *n* = 3] in the present work as compared to malonic acid [(CH_2_)n(COOH)_2,_ *n* = 1], appears to be the reason for the enhanced water uptake of the polyurethanes. It has been reported that the most stable conformer (∆G = 0) of glutaric acid in water has greater dipole moment (*p*_glutaric_ = 4.66 D) than that of malonic acid conformer in water (*p*_glutaric_ = 3.68 D) [70]. Glutaric acid has a greater dipole moment, indicating that the hydrogen bond acceptor C=O has higher electronegativity than that of malonic acid, hence polyurethanes comprising of glutaric acid in the soft segment are hypothesized to have stronger hydrogen bonding energy, thus exhibiting superior water uptake during the enzymatic degradation test (Figure 9). The higher water uptake of the polyurethane appears to be the main ground govern the superior mass loss properties of the polyurethane in the present work. Besides, the porosity also plays a role in water absorption, and this can be confirmed by higher porosity of the present polyurethanes (92–94%) as compared to the polyurethanes reported in the previous work (89–90%).

#### 3.6.3. pH Measurement

The pH of the enzymatic medium was measured and recorded averagely 6.1–6.5 after 29 days of incubation. The slight decrease in pH value in enzymatic medium after 28 days of degradation could be due to the diffusion and dissolution of acidic degradation products during enzymatic degradation [71]. Anyway, the pH changes of the incubation medium were insignificant, which could be due to the neutralization of acidic degradation products by the alkaline amino groups of hard segments [13]. Retaining the pH of the incubation medium is an important requirement for biomaterial used as scaffold or implantable material, since inflammatory responses in vivo could be reduced.

#### 3.6.4. ATR-FTIR Spectroscopy

The degradation of polyurethanes is mainly through the cleavage of ester, urethane and urea linkages, hence ATR-FTIR analysis was used to determine the chemical changes of the polyurethanes after enzymatic degradation. The intensity of the –OH stretching vibration at 3311–3363 cm^−1^ in the FTIR spectra of all the degraded samples (PU 0.8–PU 1.2) was found to be increased after the enzymatic degradation test as compared to the FTIR spectra of the samples before degradation test (Figure 5a,b).

This was mainly due to the hydrolysis of ester bonds in polyester to form the degraded products with hydroxyl groups [13]. Besides, the intensity of the OH band of degraded samples PU 1.1 and PU 1.2 was much prominent as compared to other degradation samples (PU 0.8–PU 1.0). This observation agreed with the fast degradation rate of PU 1.1 (Mass loss = 59% on day 28) and PU 1.2 (Mass loss = 51% on day 17). The –C=O stretching of ester at 1731–1717 cm^−1^ of the samples had also shown a lower intensity after the enzymatic degradation, supporting the hypothesis that the ester groups in the soft segment of the polyurethanes were cleaved into hydroxyls and carboxylic acids. The –C=O stretching vibration of ester was also observed to split into two peaks with the appearance of a new peak at 1651–1655 cm^−1^, evidencing the presence of CO^2−^ asymmetric stretching vibration resulting from the formation of carboxylic acid salts [72]. The formation of carboxylic salts could be attributed to the reaction between the carboxylic acid with the basic phosphate salt in the PBS [12]. Meanwhile, the increased intensity of the –NH bending in urethane and urea linkages at 1528–1546 cm^−1^ had evident the deformation of urethane and urea linkages in the amorphous areas of the polyurethane [63].

#### 3.6.5. Thermal Analysis

The polyurethane samples prepared with different amounts of IPDI (PU 0.8–PU 1.2) treated in enzymatic PBS for 28 days were collected for thermal analysis. All the polyurethane samples showed only two decomposition stages at T_max2_ ranged 307–341 °C and T_max3_ ranged 391–403 °C, respectively (Table 4). The decomposition step of the volatile compounds at T_max1_ was absent in the polyurethane samples after degradation process could be attributed to the loss of volatile compounds such as water and carbon dioxide during the degradation study. Both T_max2_ and T_max3_ were found to be higher in polyurethanes underwent degradation test. Besides, there were residues left after the TGA analysis, in which residues were not seen in all polyurethane samples before the degradation test. The increased decomposition temperatures for both stages (T_max2_) and (T_max3_) and residues left after the thermal decomposition at 800 °C indicated that the polyurethanes had higher thermal stability caused by changes in the polymer structure occurred during enzymatic degradation [20,60]. During the enzymatic degradation, the urethane linkages were potentially dissociated into diisocyanates and polyols [73]. The reactive diisocyanates might dimerize to form carbodiimide and carbon dioxide or undergoing trimerization to form isocyanurates or further react with water to produce the aromatic amines and carbon dioxide. The carbodiimide is unstable and could potentially react with water to form the thermally stable urea. All these potential reactions could stabilize the structure of the polyurethanes up to 400 °C [35].

#### 3.6.6. Physical Appearance and Morphology

**Figure 10:**
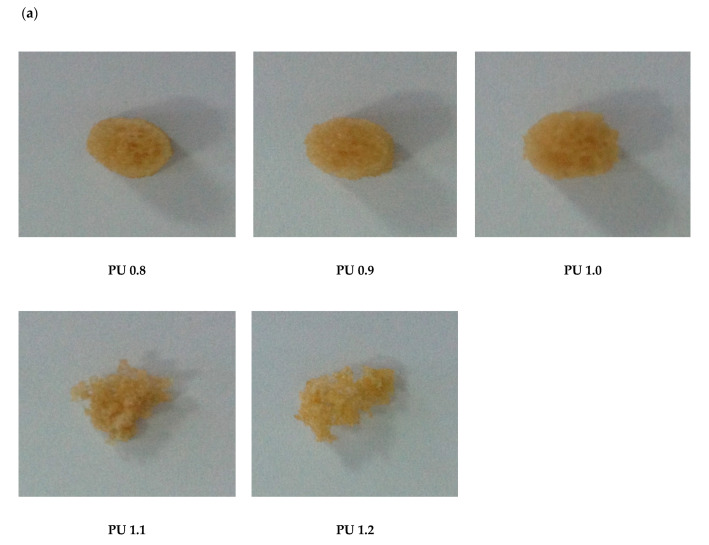

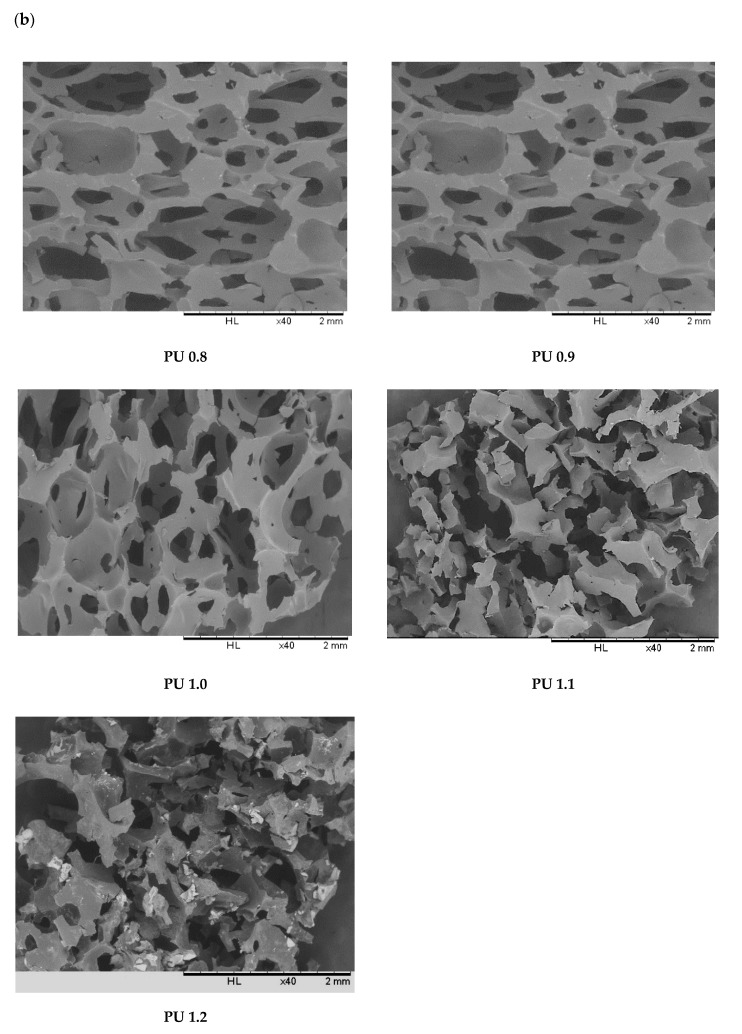
Morphology of polyurethanes after 4 weeks of enzymatic degradation study (**a**) physical appearance (**b**) SEM images (40× magnification) PU 0.8, PU 0.9, PU 1.0, PU 1.1, PU 1.2 were prepared by reacting palm oil-based polyester polyol with IPDI at an isocyanate index of 0.8, 0.9, 1.0, 1.1 and 1.2, respectively.

The physical appearance and SEM images of the polyurethanes formulated by different isocyanate indices after 4 weeks of enzymatic degradation were recorded (Figure 10a,b). PU 0.8, PU 0.9 and PU 1.0 were remained unchanged of its cylindrical shape however the surface of the polyurethanes was becoming uneven. On the other hand, PU 1.1 (28 days) and PU 1.2 (17 days) were fragmented in the enzymatic medium and lost its original shape. Through the SEM images, the internal structure of the polyurethanes was found collapsed and appearing with cracks in the polymer structure (Figure 10b).

### 3.7. Cytotoxicity by MTT Assay

**Figure 11:**
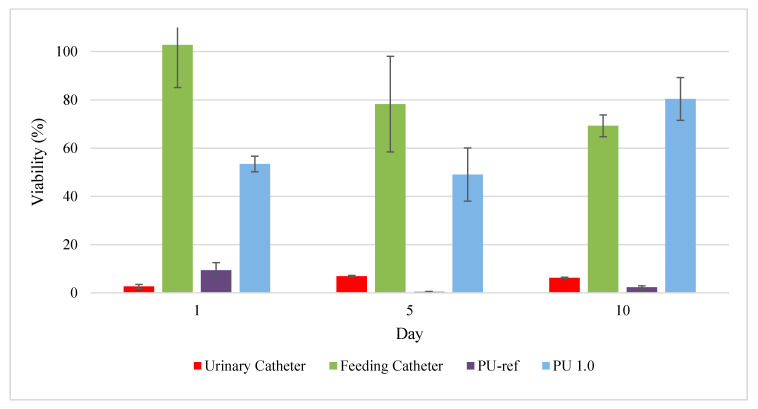
Cell viability of urinary catheter, feeding catheter, PU-ref and PU 1.0 for 1, 5 and 10 days (Mean ± SD, *n* = 9) PU-ref was prepared by reacting PCL-diol and PEG with IPDI at isocyanate index of 1.0, while PU 1.0 was prepared by reacting palm oil-based polyester polyol with IPDI at isocyanate index of 1.0.

MTT assay was used to evaluate the toxicity of the polyurethanes through the determination of the cell mitochondrial function [74,75,76]. MG-63 human osteosarcoma bone fibroblasts had been used to determine the cytotoxicity effect of the polyurethanes for tissue engineering applications [35,77]. The positive controls used in this study were feeding catheter while the negative control was urinary catheter and a reference polyurethane (PU-ref) produced by the commercial polyols, PCL diol 2000, PEG 6000 and IPDI was used. PCL diol and PEG were chosen to make comparison with PPP because they are the most used commercial polyols for the production of biodegradable and biocompatible polyurethanes. Polymeric material is known as no cytotoxicity effect if its cell viability is more than 90%, slight cytotoxicity effect for a cell viability of 60–90%, moderate cytotoxicity effect for cell viability ranges of 30–60%, and severe cytotoxicity effect for a cell viability of less than 30% [66]. On the first day of incubation, the positive control feeding catheter exhibited a non-cytotoxic effect with 102.74% of viability, while the negative control, urine catheter and PU-ref showed a severe cytotoxic effect, with 2.66% and 9.4% of viability, respectively (Figure 11). Overall, the cytotoxicity of all the controls was not improved after 10 days of incubation, where the urine catheter and PU-ref remained as severe cytotoxicity with cell viabilities of 6.23% and 2.29%, respectively. Meanwhile, the cell viability of the positive control feeding catheter was reduced to slight cytotoxicity (69%). On the other hand, PU 1.0 showed a moderate cytotoxicity effect, with 53.43% viability after 1 day of incubation. However, after 10 days of incubation, PU 1.0 demonstrated slight cytotoxicity with 80.37% of cell viability. The increased cell viability indicated the enhancement of the polyurethane biocompatibility, which could be due to the neutralization of the acidic products from the hydrolysis of ester linkages with amine by-products in the hard segment of polyurethanes [55], and hence the reduced toxicity effect. Barrioni et al. reported that the biodegradable HDI-based polyurethane film produced for biomaterial applications displayed moderate cytotoxicity to human osteoblast (SAOS), with a cell viability of 54.3% after 24 h of treatment [10]. In addition, Miao et al. reported on the soybean oil-based polyurethane biomaterial cultured with L-292 cells had demonstrated moderate cytotoxicity with a cell viability range of 46–58% after 48 h of incubation [72].

### 3.8. Cell Adhesion

**Figure 12:**
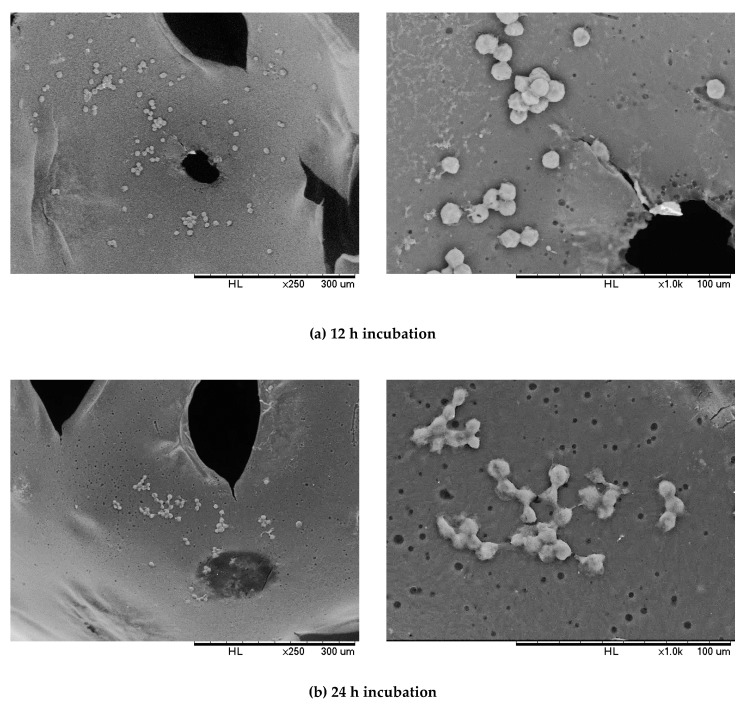
SEM images of MG-63 cells adhesion on PU 1.0 after (**a**) 12 h and (**b**) 24 h of incubation PU 1.0 was prepared by reacting palm oil-based polyester polyol with IPDI at isocyanate index of 1.0.

The MG-63 cell adhesion on PU 1.0 was observed under SEM at different magnifications after 12 h and 24 h of incubation (Figure 12). After 12 h of incubation, the rounded MG-63 cells were attached onto the pore channel of the polyurethane. Meanwhile, after 24 h of incubation, the MG-63 cells started to elongate and flatten with filopodia and connected with the adjacent cells on the polyurethane. This indicated PU 1.0 was biocompatible with MG-63 cells, which allowed the adhesion, growth, and proliferation of MG-63 cells [72].

## 4. Conclusions

Palm oil-based polyester polyol with 6698 Da and a hydroxyl value of 84.50 mg KOH/g sample was successfully reacted with the aliphatic isophorone diisocyante without the incorporation of any commercial polyol to produce a polyurethane material. The polyurethanes produced were high in porosity (92–94%) with micro and macro interconnected porous structures that resulted in high water uptake properties (up to 450%)—an important criterion for biomaterials used in biomedical applications. In terms of mechanical properties, the polyurethanes produced have potential for tissue repair and tissue regeneration applications in tissue engineering. The polyurethanes produced exhibited comparable thermal stability by the TGA analysis. With regard to biodegradability, the polyurethanes prepared had a tunable degradation rate, whereby the fastest degradable polyurethane demonstrated 51% of mass loss on day 17, and the slowest degradable polyurethane degraded with 7.3% of mass loss after 1 month of enzymatic degradation study. The FTIR analysis evidenced the hydrolysis of ester and urethane linkages, while the TGA analysis proved that the chemical changes occurred during the enzymatic degradation test. It is noteworthy that the pH of the medium showed insignificant changes, while the cytotoxicity study of the selected polyurethane showed cell viability of 80.37% after 10 days of incubation. The polyurethane produced by 100% of PPP has demonstrated its potential as soft cellular porous biomaterial for tissue engineering applications.

## Figures and Tables

**Table 1:** Physico-chemical properties of palm oil-based polyester polyol.

Physico-Chemical Properties	Value	
Acid Value (mg KOH/g sample)	1.95	
Oxirane oxygen content (%)	0.35	
Hydroxyl value (mg KOH/g sample)	84.50	
M_n_	6698	
Viscosity (Pa.s)	25 °C:	24.55
	40 °C:	8.68
Pour Point (°C)	12	
Cloud Point (°C)	12	
Physical Properties	Liquid	

**Table 2:** Polyurethane formulation and PU-ref formulation.

Components	Composition (g)	
	PU	PU-Ref
PEG 6000	-	40.9
PCL diol 2000	-	9.1
PPP	50	-
A33	2	2
L580	2	2
Glycerol	3	3
DBTDL	0.4	0.2
Water	Ranged from 0.6–1.4	0.5

**Table 3:** Physical and mechanical properties of polyurethanes produced by different isocyanate indices (Mean ± SD, *n* = 3).

Polyurethane	Physical Properties			Tensile Properties			Compression Stress (kPa)
	Density (kg/m^3^)	Porosity (%)	Pore Size (μm)	Tensile Strength (kPa)	Elongation at Break (%)	Elastic Modulus (kPa)	
**PU 0.8**	96.30	92.07	37–1322	48.32 ± 8.2	76.62 ± 8.3	79.83 ± 3.4	15.10 ± 0.4
**PU 0.9**	68.00	94.44	43–1464	53.77 ± 8.6	66.83 ± 5.6	100.36 ± 53.2	16.92 ± 3.3
**PU 1.0**	87.00	92.85	43–1013	111.25 ± 4.1	59.63 ± 0.7	243.69 ± 30.2	64.08 ± 12.3
**PU 1.1**	71.60	94.13	49–1655	85.27 ± 8.5	45.86 ± 3.6	289.67 ± 58.6	49.19 ± 7.6
**PU 1.2**	71.20	94.17	57–1700	70.85 ± 13.4	32.12 ± 1.6	482.69 ± 70.3	115.09 ± 18.2

**Table 4:** Thermal analysis of polyurethanes produced by different isocyanate indices before and after enzymatic degradation study.

Polyurethane	Before Degradation								After Degradation						
	T_5%_ (°C)	T_10%_ (°C)	T_50%_ (°C)	T_90%_ (°C)	Residue at 800 °C (%)	T_max1_ (°C)	T_max2_ (°C)	T_max3_ (°C)	T_5%_ (°C)	T_10%_ (°C)	T_50%_ (°C)	T_90%_ (°C)	Residue at 800 °C (%)	T_max2_ (°C)	T_max3_ (°C)
**PU 0.8**	248	280	379	430	0	287	306	398	256	279	373	424	0.23	307	395
**PU 0.9**	250	280	373	433	0	290	311	390	258	280	373	422	0.23	308	391
**PU 1.0**	254	279	373	426	0	286	308	394	256	278	367	432	0.07	309	391
**PU 1.1**	258	280	373	428	0	285	307	395	267	285	369	429	0.34	312	395
**PU 1.2**	256	276	372	429	0	281	309	394	260	282	377	719	8.06	341	403

## References

[1] Jaganathan S., Mani M., Khudzari A. (2019). Electrospun combination of peppermint oil and copper sulphate with conducive physico-chemical properties for wound dressing applications. Polymers.

[2] Pereira Rodrigues I.C., Tamborlin L., Rodrigues A.A., Jardini A.L., Ducati Luchessi A., Maciel Filho R., Najar Lopes E.S., Gabriel L.P. (2020). Fibrous membranes tailored by rotary jet spinning for tissue engineering applications. J. Appl. Polym. Sci..

[3] Wang C., Xie J., Xiao X., Chen S., Wang Y. (2019). Development of nontoxic biodegradable polyurethanes based on polyhydroxyalkanoate and l-lysine diisocyanate with improved mechanical properties as new elastomers scaffolds. Polymers.

[4] Tajau R., Rohani R., Wan Isahak W.N.R., Salleh M.Z., Ghazali Z. (2018). Development of new bio-based polyol ester from palm oil for potential polymeric drug carrier. Adv. Polym. Tech..

[5] Li M., Chen J., Shi M., Zhang H., Ma P., Guo B. (2019). Electroactive anti-oxidant polyurethane elastomers with shape memory property as non-adherent wound dressing to enhance wound healing. Chem. Eng. J..

[6] Peng Z., Zhou P., Zhang F., Peng X. (2018). Preparation and properties of polyurethane hydrogels based on hexamethylene diisocyanate/polycaprolactone-polyethylene glycol. J. Macromol. Sci. Part B.

[7] Shahrousvand M., Mir Mohamad Sadeghi G., Salimi A. (2016). Artificial extracellular matrix for biomedical applications: Biocompatible and biodegradable poly(tetramethylene ether) glycol/poly(ε-caprolactone diol)-based polyurethanes. J. Biomater. Sci. Polym. Ed..

[8] Park J.S., Kang H.J., Lee B.-T., Choi J.S., Yim J.-H. (2020). Mechanically and electrically enhanced polyurethane-poly(3,4-ethylenedioxythiophene) conductive foams with aligned pore structures promote MC3T3-E1 cell growth and proliferation. ACS Appl. Polym. Mater..

[9] Yeganeh H., Hojati-Talemi P. (2007). Preparation and properties of novel biodegradable polyurethane networks based on castor oil and poly(ethylene glycol). Polym. Degrad. Stab..

[10] Miao S., Sun L., Wang P., Liu R., Su Z., Zhang S. (2012). Soybean oil-based polyurethane networks as candidate biomaterials: Synthesis and biocompatibility. Eur. J. Lipid Sci. Technol..

[11] Luo X., Yu Z., Cai Y., Wu Q., Zeng J. (2019). Facile fabrication of environmentally-friendly hydroxyl-functionalized multiwalled carbon nanotubes/soy oil-based polyurethane nanocomposite bioplastics with enhanced mechanical, thermal, and electrical conductivity properties. Polymers.

[12] Zieleniewska M., Auguścik M., Prociak A., Rojek P., Ryszkowska J. (2014). Polyurethane-urea substrates from rapeseed oil-based polyol for bone tissue cultures intended for application in tissue engineering. Polym. Degrad. Stab..

[13] Zieleniewska M., Leszczyński M., Kurańska M., Prociak A., Szczepkowski L., Krzyżowska M., Ryszkowska J. (2015). Preparation and characterisation of rigid polyurethane foams using a rapeseed oil-based polyol. Ind. Crop. Prod..

[14] Kong X., Liu G., Curtis J.M. (2011). Characterization of canola oil based polyurethane wood adhesives. Int. J. Adhes. Adhes..

[15] Pillai P.K.S., Li S., Bouzidi L., Narine S.S. (2018). Polyurethane foams from chlorinated and non-chlorinated metathesis modified canola oil polyols. J. Appl. Polym. Sci..

[16] Ang K.P., Lee C.S., Cheng S.F., Chuah C.H. (2014). Synthesis of palm oil-based polyester polyol for polyurethane adhesive production. J. Appl. Polym. Sci..

[17] Chuayjuljit S., Sangpakdee T., Saravari O. (2007). Processing and properties of palm oil-based rigid polyurethane foam. J. Met. Mater. Miner..

[18] Pawlik H., Prociak A. (2012). Influence of palm oil-based polyol on the properties of flexible polyurethane foams. J. Polym. Environ..

[19] Ng W.S., Lee C.S., Chuah C.H., Cheng S.F. (2017). Preparation and modification of water-blown porous biodegradable polyurethane foams with palm oil-based polyester polyol. Ind. Crop. Prod..

[20] Somarathna H., Raman S., Badri K., Mutalib A., Mohotti D., Ravana S. (2016). Quasi-static behavior of palm-based elastomeric polyurethane: For strengthening application of structures under impulsive loadings. Polymers.

[21] Badri K.H., Othman Z., Ahmad S.H. (2004). Rigid polyurethane foams from oil palm resources. J. Mater. Sci..

[22] Liu J., Yang Y., Gao B., Li Y.C., Xie J. (2019). Bio-based elastic polyurethane for controlled-release urea fertilizer: Fabrication, properties, swelling and nitrogen release characteristics. J. Clean. Prod..

[23] Yaakob Z., Min A.M., Hilmi M.M., Zaman H.M.D.K., Kamarudin S.K.K. (2009). Effect of compactabilization of polymer on the properties of polyurethane-palm fiber composites. J. Polym. Eng..

[24] Hassouna Y.M., Somayeh Z.K., Kafienahb W., Husam M.Y. (2018). Synthesis, characterization & cytocompatibility of poly(diol-co-tricarballylate) based thermally crosslinked elastomers for drug delivery & tissue engineering applications. Mater. Sci. Eng. C.

[25] Yang J., Antonio R.W., Samuel J.P., Hageman G., Guillermo A.A. (2006). Synthesis and evaluation of poly(diol citrate) biodegradable elastomers. Biomaterials.

[26] Mani M.P., Jaganathan S.K., Prabhakaran P., Nageswaran G., Krishnasamy N.P. (2019). Electrospun polyurethane patch in combination with cedarwood and cobalt nitrate for cardiac applications. J. Appl. Polym. Sci..

[27] Molina G.A., Elizalde-Mata A., Hernández-Martínez Á.R., Fonseca G., Cruz Soto M., Rodríguez-Morales Á.L., Estevez M. (2020). Synthesis and characterization of inulin-based responsive polyurethanes for breast cancer applications. Polymers.

[28] Jaganathan S.K., Mani M.P. (2019). Single-stage synthesis of electrospun polyurethane scaffold impregnated with zinc nitrate nanofibers for wound healing applications. J. Appl. Polym. Sci..

[29] Xue L., Greisler H.P. (2003). Biomaterials in the development and future of vascular grafts. J. Vasc. Surg..

[30] Pfister D.P., Xia Y., Larock R.C. (2011). Recent advances in vegetable oil-based polyurethanes. ChemSusChem.

[31] Yeoh F.H., Lee C.S., Kang Y.B., Wong S.F., Cheng S.F. (2018). One-pot synthesis of palm oil-based polyester polyol for production of biodegradable and biocompatible polyurethane. J. Appl. Polym. Sci..

[32] Skrobot J., Ignaczak W., el Fray M. (2015). Hydrolytic and enzymatic degradation of flexible polymer networks comprising fatty acid derivatives. Polym. Degrad. Stab..

[33] Song N., Jiang X., Li J., Pang Y., Li J., Tan H., Fu Q. (2013). The degradation and biocompatibility of waterborne biodegradable polyurethanes for tissue engineering. Chin. J. Polym. Sci..

[34] Rottmar M., Richter M., Mäder X., Grieder K., Nuss K., Karol A., Rechenberg B., Zimmermann E., Buser S., Dobmann A. (2015). In vitro investigations of a novel wound dressing concept based on biodegradable polyurethane. Sci. Tech. Adv. Mater..

[35] Barrioni B.R., de Carvalho S.M., Oréfice R.L., de Oliveira A.A.R., Pereira M.D.M. (2015). Synthesis and characterization of biodegradable polyurethane films based on hdi with hydrolyzable crosslinked bonds and a homogeneous structure for biomedical applications. Mater. Sci. Eng C.

[36] Aastha S.D., Thomas S., Rane A.V., Kanny K., Abitha V.K., Thomas M.G. (2018). Polyurethane foam chemistry. Recycling of Polyurethane Foams.

[37] Pinchuk L. (1995). A review of the biostability and carcinogenicity of polyurethanes in medicine and the new generation of “biostable” polyurethanes. J. Biomater. Sci., Polym. Ed..

[38] Gabriel L., Zavaglia C., Jardini A., Dias C., Maciel Filho R. (2014). Isocyanates as precursors to biomedical polyurethanes. Chem. Eng. Trans..

[39] Zhang X.D., Macosko C.W., Davis H.T., Nikolov A.D., Wasan D.T. (1999). Role of silicone surfactant in flexible polyurethane foam. J. Colloid Interface Sci..

[40] Kim S.H., Kim B.K., Lim H. (2008). Effect of isocyanate index on the properties of rigid polyurethane foams blown by HFC 365mfc. Macromol. Res..

[41] Dworakowska S., Bogdal D., Prociak A. (2012). Microwave-assisted synthesis of polyols from rapeseed oil and properties of flexible polyurethane foams. Polymers.

[42] Gogolewski S., Gorna K., Turner A.S. (2006). Regeneration of bicortical defects in the iliac crest of estrogen-deficient sheep, using new biodegradable polyurethane bone graft substitutes. J. Biomed. Mater. Res. Part A.

[43] Polo-Corrales L., Latorre-Esteves M., Ramirez-Vick J.E. (2014). Scaffold design for bone regeneration. J. Nanosci. Nanotechnol..

[44] Kucinska-Lipka J., Marzec M., Gubanska I., Janik H. (2017). Porosity and swelling properties of novel polyurethane–ascorbic acid scaffolds prepared by different procedures for potential use in bone tissue engineering. J. Elastom. Plast..

[45] Spaans C., Belgraver V., Rienstra Ode Groot J., Veth R., Pennings A. (2000). Solvent-free fabrication of micro-porous polyurethane amide and polyurethane-urea scaffolds for repair and replacement of the knee-joint meniscus. Biomaterials.

[46] Kucińska-Lipka J., Gubanska I., Skwarska A. (2017). Microporous polyurethane thin layer as a promising scaffold for tissue engineering. Polymers.

[47] Hafeman A., Yoshii B., Zienkiewicz T., Davidson K.J., Guelcher S. (2008). Injectable biodegradable polyurethane scaffolds with release of platelet-derived growth factor for tissue repair and regeneration. Pharm. Res..

[48] Dong Z., Li Y., Zou Q. (2009). Degradation and biocompatibility of porous nano hydroxyapatite/polyurethane composite scaffold for bone tissue engineering. Appl. Surf. Sci..

[49] Lu Y., Larock R.C. (2008). Soybean-oil-based waterborne polyurethane dispersions: Effects of polyol functionality and hard segment content on properties. Biomacromolecules.

[50] Asensio M., Costa V., Nohales A., Bianchi O., Gómez C.M. (2019). Tunable structure and properties of segmented thermoplastic polyurethanes as a function of flexible segment. Polymers.

[51] Wang K., Peng Y., Tong R., Wang Y., Wu Z. (2010). The effects of isocyanate index on the properties of aliphatic waterborne polyurethaneureas. J. Appl. Polym. Sci..

[52] Kattiyaboot T., Thongpin C. (2016). Effect of natural oil based polyols on the properties of flexible polyurethane foams blown by distilled water. Energy Procedia.

[53] Prociak A., Malewska E., Bąk S. (2016). Influence of isocyanate index on selected properties of flexible polyurethane foams modified with various bio-components. J. Renew. Mater..

[54] Fan H., Tekeei A., Suppes G.J., Hsieh F.H. (2012). Physical properties of soy-phosphate polyol-based rigid polyurethane foams. Int. J. Polym. Sci..

[55] Bil M., Ryszkowska J., Kurzydłowski K.J. (2009). Effect of polyurethane composition and the fabrication process on scaffold properties. J. Mater. Sci..

[56] Modesti M., Lorenzetti A. (2001). An experimental method for evaluating isocyanate conversion and trimer formation in polyisocyanate–polyurethane foams. Eur. Polym. J..

[57] Rojek P., Prociak A. (2012). Effect of different rapeseed-oil-based polyols on mechanical properties of flexible polyurethane foams. J. Appl. Polym. Sci..

[58] Gorna K., Gogolewski S. (2003). Preparation, degradation, and calcification of biodegradable polyurethane foams for bone graft substitutes. J. Biomed. Mater. Res..

[59] Das B., Konwar U., Mandal M., Karak N. (2013). Sunflower oil based biodegradable hyperbranched polyurethane as a thin film material. Ind. Crop. Prod..

[60] Ryszkowska J.L., Auguścik M., Sheikh A., Boccaccini A.R. (2010). Biodegradable polyurethane composite scaffolds containing Bioglass^®^ for bone tissue engineering. Compos. Sci. Technol..

[61] Cangemi J.M., Claro Neto S., Chierice G.O., Santos A.M.D. (2006). Study of the biodegradation of a polymer derived from castor oil by scanning electron microscopy, thermogravimetry and infrared spectroscopy. Polímeros.

[62] Javni I., Petrovi Z.S., Guo A., Fuller R. (2000). Thermal stability of polyurethanes based on vegetable oils. J. Appl. Polym. Sci..

[63] Gómez E.F., Luo X., Li C., Michel F.C., Li Y. (2014). Biodegradability of crude glycerol-based polyurethane foams during composting, anaerobic digestion and soil incubation. Polym. Degrad. Stab..

[64] Wang J., Zheng Z., Wang Q., Du P., Shi J., Wang X. (2013). Synthesis and characterization of biodegradable polyurethanes based on L-cystine/cysteine and poly(ϵ-caprolactone). J. Appl. Polym. Sci..

[65] Prociak A., Rojek P., Pawlik H. (2012). Flexible polyurethane foams modified with natural oil based polyols. J. Cell. Plast..

[66] Norouz F., Halabian R., Salimi A., Ghollasi M. (2019). A new nanocomposite scaffold based on polyurethane and clay nanoplates for osteogenic differentiation of human mesenchymal stem cells in vitro. Mater. Sci. Eng. C.

[67] Tawagi E., Ganesh T., Cheng H.L.M., Santerre J.P. (2019). Synthesis of degradable-polar-hydrophobic-ionic co-polymeric microspheres by membrane emulsion photopolymerization: In vitro and in vivo studies. Acta Biomater..

[68] Shen Z., Lu D., Li Q., Zhang Z., Zhu Y. (2015). Synthesis and characterization of biodegradable polyurethane for hypopharyngeal tissue engineering. BioMed Res. Int..

[69] Pan Z., Ding J. (2012). Poly(lactide-*co*-glycolide) Porous scaffolds for tissue engineering and regenerative medicine. Interface Focus..

[70] Nguyen T.H., Hibbs D.E., Howard N.T. (2005). Conformations, energies, and intramolecular hydrogen bonds in dicarboxylic acids: Implications for the design of synthetic dicarboxylic acid receptors. J. Comput. Chem..

[71] Wang Z., Yu L., Ding M., Tan H., Li J., Fu Q. (2011). Preparation and rapid degradation of nontoxic biodegradable polyurethanes based on poly(lactic acid)-poly(ethylene glycol)-poly(lactic acid) and l-lysine diisocyanate. Polym. Chem..

[72] Guelcher S., Srinivasan A., Hafeman A., Gallagher K., Doctor J., Khetan S., McBride S., Hollinger J. (2007). Synthesis, in vitro degradation, and mechanical properties of two-component poly(ester urethane)urea scaffolds: Effects of water and polyol composition. Tissue Eng..

[73] Bugajny M., le Bras M., Bourbigot S. (2000). Thermoplastic polyurethanes as carbonization agents in intumescent blends. Part 2: Thermal behavior of polypropylene/thermoplastic polyurethane/ammonium polyphosphate blends. J. Fire Sci..

[74] Choi H., Lee J., Lee J., Sung H., Shin J., Shin J., Wu Y., Kim J. (2016). MG-63 cells proliferation following various types of mechanical stimulation on cells by auxetic hybrid scaffolds. Biomater. Res..

[75] Encalada-Diaz I., Cole B., MacGillivray J., Ruiz-Suarez M., Kercher J., Friel N., Valero-Gonzalez F. (2011). Rotator cuff repair augmentation using a novel polycarbonate polyurethane patch: Preliminary results at 12 months’ follow-up. J. Shoulder Elb. Surg..

[76] Wang L., Li Y., Zuo Y., Zhang L., Zou Q., Cheng L., Jiang H. (2009). Porous bioactive scaffold of aliphatic polyurethane and hydroxyapatite for tissue regeneration. Biomed. Mater..

[77] Lönnroth E.C. (2005). Toxicity of medical glove materials: A pilot study. Int. J. Occup. Saf. Ergon..

